# Light as a Modulator of Non-Image-Forming Brain Functions—Positive and Negative Impacts of Increasing Light Availability

**DOI:** 10.3390/clockssleep5010012

**Published:** 2023-03-17

**Authors:** Islay Campbell, Roya Sharifpour, Gilles Vandewalle

**Affiliations:** GIGA-Cyclotron Research Centre-In Vivo Imaging, University of Liège, 4000 Liège, Belgium; icampbell@uliege.be (I.C.); roya.sharifpour@uliege.be (R.S.)

**Keywords:** melanopsin, non-image forming, circadian rhythms, cognition, blue light, functional magnetic resonance imaging, intrinsically photosensitive retinal ganglion cells, teenagers, ageing, sleep

## Abstract

Light use is rising steeply, mainly because of the advent of light-emitting diode (LED) devices. LEDs are frequently blue-enriched light sources and may have different impacts on the non-image forming (NIF) system, which is maximally sensitive to blue-wavelength light. Most importantly, the timing of LED device use is widespread, leading to novel light exposure patterns on the NIF system. The goal of this narrative review is to discuss the multiple aspects that we think should be accounted for when attempting to predict how this situation will affect the NIF impact of light on brain functions. We first cover both the image-forming and NIF pathways of the brain. We then detail our current understanding of the impact of light on human cognition, sleep, alertness, and mood. Finally, we discuss questions concerning the adoption of LED lighting and screens, which offer new opportunities to improve well-being, but also raise concerns about increasing light exposure, which may be detrimental to health, particularly in the evening.

## 1. Introduction

There are two light-sensitive photoreceptor pathways in the human retina. First, the classical visual system is required for image formation and relies on rod and cone photoreceptors. Second, the non-image forming (NIF) system, also referred to as the “non-visual” system, detects environmental irradiance [1,2]. The main photoreceptor of the NIF system was discovered only about two decades ago, termed intrinsically photosensitive retinal ganglion cells (ipRGCs) due to the expression of the photopigment melanopsin, which is maximally sensitive to blue-wavelength light around 480 nm [2,3,4]. Melanopsin-expressing ipRGCs mediate the influence of light on several circadian, neuroendocrine, and neurobehavioral functions collectively defined as NIF, i.e., functions not directly related to image formation. Light can have acute impacts on NIF functions including melatonin suppression, pupillary constriction, and stimulation of alertness and cognitive performance [5,6,7]. On a longer timescale, light can affect circadian entrainment and influence mood [8,9]. 

Light is now emerging as being central to our health and well-being, and several health issues have been associated with unhealthy light environments including sleepiness, cognitive impairments, mood, and sleep disorders [8,10]. The development of light-emitting diode (LED) lighting was a major technological advance that was awarded the 2014 Nobel Prize for Physics [11] and has turned light into a truly tuneable parameter. However, with LEDs being easily incorporated into many devices, light use has expanded. Moreover, many commonly used white LEDs are relatively rich in blue-wavelength light [12]. 

This narrative literature review discusses the multiple aspects that we think should be considered to predict the impact of modern, changing light environments on brain functions. We include what we think are important and relevant papers to cover the relatively broad topics of this review, but we cannot be exhaustive and are inherently subjective in our selection. We first provide an overview of the retinal and neural light-sensitive pathways and our current understanding of light’s effect on cognition, sleep, alertness, and mood. We also discuss the potential biological impacts of increasing LED lighting and take into consideration other questions including lifetime changes from adolescence to senescence, light’s impact on mood and emotional regulation, and the confusion surrounding light’s impact on the retina. 

## 2. Current ‘Modern’ Lighting

**Figure 1:**
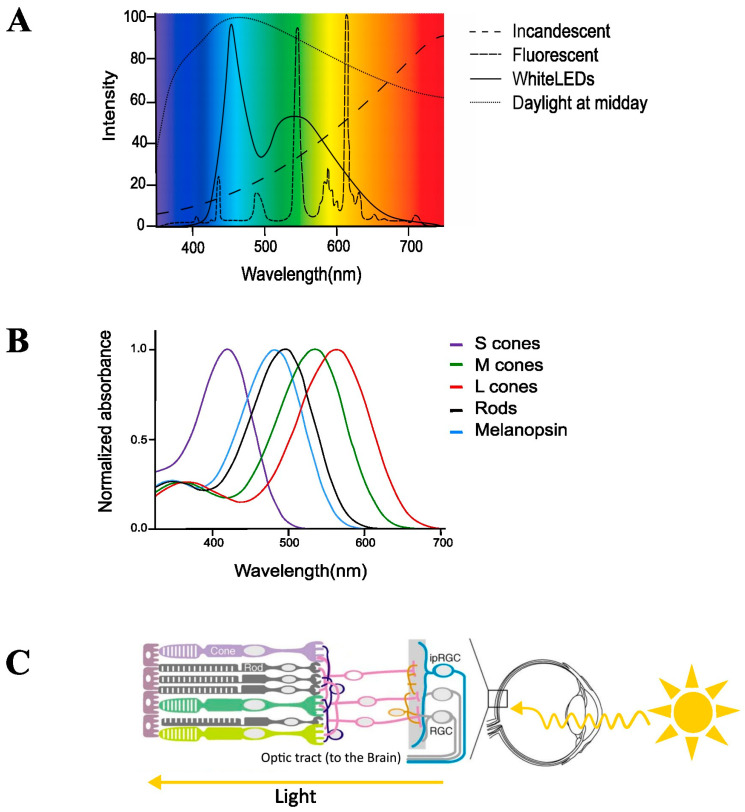
Photoreceptor sensitivities and light spectra. (**A**) Spectrum of white LED, fluorescent, and incandescent light sources and natural daylight. (**B**) Spectral sensitivities of retinal photoreceptors in primates. (**C**) Wiring and position of retinal photoreceptors. ipRGCs: intrinsically photosensitive retinal ganglion cells expressing melanopsin. RGC: retinal ganglion cells. Reproduced and adapted with permission from [14].

White LEDs were first developed in 1996 [11] but they have been adopted worldwide due to their falling prices, improved lighting qualities, and lower energy consumption. There are different ways to produce white LEDs, but the most common method is by combining a blue LED and yellow phosphors, which absorb part of the blue light to emit longer wavelength photons, producing light that appears white [13]. These common “white” LEDs are typically blue-enriched light sources with a peak around 440–460 nm, which falls within the sensitivity range of the NIF system, and a second broader peak in the yellow–green wavelength region (Figure 1A). This emission spectrum is very different from incandescent and fluorescent lights, which have a dominant wavelength closer to the sensitivity of the classical visual system (550 nm) [2]. The advent of incandescent lighting has led to blue-depleted indoor light exposure but the conversion to LED lighting means we are now becoming increasingly exposed to more blue-wavelength light. However, whether this change in the spectral composition of light sources translates to a differential impact on NIF functions is still being determined. 

What is known is that light’s impact depends on the timing and duration of the exposure, meaning that more blue-wavelength (~460–480 nm) light can have beneficial or detrimental effects, including changes in alertness and cognition [8,15]. Governments worldwide are adopting policies in favour of LED lighting. Lighting industries are even proposing LED-integrative lighting products to improve health, mood, or well-being, often with little solid scientific backing [16]. LED lighting is now found everywhere in our homes and offices, and LED back-lighted screen displays are now found in computers, televisions, phones, tablets, etc. As detailed in the review, it may not be the spectrum of LEDs but rather the timing of their use that is most problematic. The use of LEDs is expected to continue to rise rapidly worldwide in the coming decades [13], making the understanding of the NIF impact of light a timely research question. 

## 3. Classical Light-Sensitive Pathways of the Brain for the Visual Systems

Rods and cones densely populate the photoreceptor outer layer of the retina. They are sensitive to light due to having specialized stacked membranes that contain high concentrations of photopigments [1]. Rods are required for scotopic night vision as they can detect very low amounts of photons and they express the photopigment rhodopsin, which has a peak sensitivity at 507 nm [2]. Scotopic vision is colour-blind as there is only a single type of rod. In humans, photopic vision is mediated by three different cone photoreceptors, each with different peak wavelength sensitivities, enabling colour vision. Short-wavelength cones (S-cones) express opsin cyanolabe and have a peak sensitivity around 420 nm; mid-wavelength cones express chlorolabe opsin and are most sensitive around 535 nm photons; and long wavelength-cones express erythrolabe with a peak sensitivity around 565 nm (peak values may vary slightly depending on pre-retinal filtering). This results in an overall maximal photopic sensitivity over the yellow–green part of the visible light spectrum (~550 nm) [2]. Cones are insensitive to scotopic light levels (~10–6 Cd/m^2^) and rod saturation begins at photopic light levels (~10 Cd/m^2^). Between scotopic and photopic lies mesoscopic vision with rod and cone contribution to (partially coloured) vision [17]. Following signal processing by amacrine, horizontal and bipolar cells, rods, and cones signal and then reach the retinal ganglion cell (RGC) layer. A large number of RGC types have been isolated with different wavelengths and spatial opponency, which shape their overall axonal response in the optic nerve [1]. Importantly, these RGCs typically respond immediately to light in a time-locked manner. The subcortical brain areas innervated by classical photoreceptors include the thalamic lateral geniculate nucleus (LGN) before reaching occipital areas involved in complex image formation, but also the superior colliculus and the lateral posterior pulvinar complex [18].

## 4. Non-Image-Forming System

The prediction of a second novel photoreceptor system within the mammalian eye was first made in 1927 by Keeler, noting that “apparently blind” mice still maintained pupil constriction when exposed to light [19]. This prediction would not be considered seriously until about 50 years later when rodent animal models with complete enucleation were reported to lose a NIF function and photoentrainment could not be explained by the photoreceptors of the classical visual system [20,21]. The later development of mouse models genetically engineered to completely lack rods and cones allowed for true testing of Keeler’s prediction. These mouse models exhibited NIF responses to light, such as pineal melatonin suppression, pupillary light reflex, and circadian entrainment, with a maximal sensitivity towards the shorter wavelengths [22,23,24]. Furthermore, the retinal hypothalamic tract remained intact in “rod/coneless” mice, projecting to suprachiasmatic nuclei (SCN), olivary pretectal nuclei (OPN), and inter-geniculate leaflet regions (known to be involved in circadian entrainment and NIF responses) [25].

In humans, Czeisler et al. reported in the 1990s that a completely blind individual retained melatonin suppression by light [26]. Later studies of colour-blind subjects suggested that deficiencies in any of the cone types had no detectable impact on melatonin suppression by light [27]. Two studies further investigated the spectral sensitivity of melatonin suppression in humans with normal sight and these studies identified the shorter wavelength region of the visual spectrum (446–477 nm) as having the greatest impact on melatonin suppression [6,28].

The NIF system was discovered to be mainly driven by melanopsin-expressing ipRGCs, a third class of retinal photoreceptors [2,4]. Melanopsin was first discovered in batrachian skin right before the turn of the millennium and then later identified in mammalian retinas [29]. Melanopsin-expressing ipRGCs only make up around 5% and 1% of all retinal ganglion cells in mouse and human retina, respectively, and these photoreceptors measure environmental irradiance [2]. The difference in melanopsin-expressing ipRGCs between mice and humans may be due to different methodologies used, with human ipRGCs studies unable to use the most sensitive techniques. The blue-sensitive melanopsin photopigment is encoded by the OPN4 gene [3]. Longer wavelengths, such as red light (>~600 nm), have a largely reduced effect on the photopigment light transducing form. Animal and human studies confirmed that melanopsin is the main photopigment of the NIF system, shifting its sensitivity towards short-wavelength light, around 480 nm (Figure 1B) [6,24,28,30].

Melanopsin is a dual-state photopigment, meaning it exists in two stable photon absorption states, driving phototransduction and chromophore regeneration, respectively, similar to rhabdomomeric photopigments of invertebrates [31,32,33]. This is in contrast to rod and cone photopigments where photons drive the phototransduction while chromophore regeneration requires an enzyme cycle taking place in the nearby cells of the retinal pigment epithelium [31]. The conversion of melanopsin between its 11-cis and all-trans isoforms is driven by different light wavelengths with 480 nm photons most efficient in the 11-cis-to-all-trans switch triggering phototransduction, while the all-trans-to-11-cis reconversion takes place at longer wavelengths, subject to debate [31,32]. Biochemistry investigation reported that chromophore regeneration maximal sensitivity lies only about 10 nm away from the peak of phototransduction efficiency [33]. In contrast, in vivo studies in mice and humans suggest that orange/reddish wavelength light (590–620 nm) most efficiently drives chromophore regeneration and leads to the subsequent increase in intrinsic photosensitivity of ipRGCs [31,32]. Increased sensitivity following longer wavelength light may depend on the particular in-lab protocol (including periods of complete darkness and different light levels) as studies combining blue (479 nm) and red (627 nm) light LEDs failed to modulate light’s impact [34].

Contrary to the initial predictions, the classical and NIF systems are not separate. Melanopsin-ipRGCs innervate the LGN and, indirectly, the primary visual cortex. They are involved in some important visual functions such as brightness detection, rod/cone light level adaptation (contributing to the remarkable 10^12^ fold change our vision operates over), and they were reported to contribute to coarse image formation and spatial contrast detection [35,36,37,38]. Further roles of melanopsin-ipRGCs contributing to visual functions include improved visual information processing in the retina and dLGN through the modulation of fast narrowband oscillations; maintaining a generalized increase in neural activity in response to changing background light intensity; and increasing the firing rate in the optic nerve due to changes in ambient light level [39,40,41].

IpRGCs also receive input from rods and cones, which is required for complete NIF responses (Figure 1C) [42]. For instance, rods and cones contribute to the phasic pupil light reflex (PLR) at continuous and lower light intensities, whereas melanopsin mainly contributes to the PLR at higher light intensities and sustains the PLR for longer durations [7]. Importantly, melanopsin-driven photoreception outlasts light exposure from seconds to tens of minutes after lights off. These characteristics of ipRGCs drive so-called post-illumination pupil constriction, an “after-effect” constriction, that offers a unique means to directly measure melanopsin function in humans [43]. 

**Figure 2:**
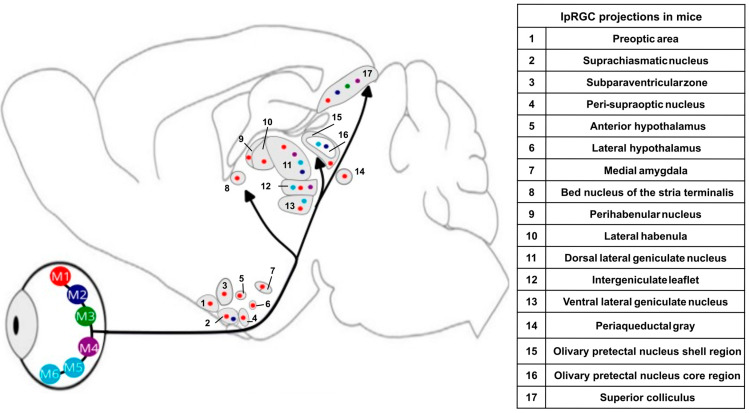
Schematic of main ipRGC projections in mice. Adapted with permission from [44,48]. Information from [36,44,46,48]. Numbers of the scheme correspond to numbers in the adjacent table. Coloured dots correspond to known projection of ipRGCs subtypes in rodents (M1 to M6).

Rodent studies have established that melanopsin ipRGCs are composed of at least six different subtypes (to date: M1–M6) determined based on morphological and functional features. IpRGC subtypes have varying levels of melanopsin expression, complex interactions with rods and cones, and different projection patterns to subcortical brain regions (Figure 2) [44]. M1 ipRGCs are currently the best-defined subtype. They have the highest level of melanopsin photopigment expression and densely innervate the SCN, the site of the master circadian clock (making up 80% of total SCN ipRGC innervation) [45]. M1 ipRGCs appear to be the main subtype required for encoding environmental irradiance [46]. The M1 ipRGCs also project to the OPN, driving the pupil light reflex; the perihabenular zone, involved in mood regulation; the intergeniculate leaflet, involved in the circadian response to light; the lateral hypothalamus, important for sleep and wakefulness regulation; the visual ventral LGN; and other subcortical areas, but with a reduced innervation density [44,45]. Furthermore, using a different genetic labelling technique, ipRGCs were found to project to the central amygdala, zona incerta, and the accessory optic system [47].

The other ipRGC subtypes are less well-defined. M2 ipRGCs make up the other 20% of the retinal input to the SCN. M2 ipRGCs may also play a role in pupil constriction, as they contribute 55% of ipRGC innervations to the OPN [45]. Recently, M4 ipRGC subtypes have been implicated in a multi-synaptic pathway involved in mood regulation [49]. Other non-M1 ipRGC subtypes are known to contribute to visual perception [50]. The exact roles of each subtype in NIF and visual functions are still being elucidated.

Importantly, the melanopsin-driven light response is considered to be sluggish, but this initial observation depends on the ipRGC subtype and light levels. M1 peak responses are detected within a second while M4 peak responses can take up to 20 s [46]. When functional rods and cones are present, ipRGCs respond immediately to light [42]. It is worth mentioning that it is believed that ipRGCs primarily drive NIF functions via the release of excitatory neurotransmitters at NIF brain targets, but there is a subset of ipRGCs in mice that release inhibitory neurotransmitters (GABA), through which some non-image-forming behaviours, such as pupillary reflex and circadian entrainment become relatively insensitive at low light levels [51]. There is growing research on human ipRGCs with potentially four ipRGC subtypes identified. However, studies on human ipRGCs are scarce and need to be replicated, and many unanswered questions remain about the differences between mouse ipRGCs and human ipRGCs (see review [52]). 

There is evidence of other photoreceptors contributing to the NIF system. S-cone photoreceptors seem to contribute to circadian photoentrainment through a blue–yellow colour discrimination circuit involving M1-ipRGCs. It is proposed that colour opponency evolved to distinguish between the different sky colours encountered at different times of the day, to convey timing information to the SCN and to support correct entrainment [53,54,55]. In mice, there is some evidence that cones also contribute to measuring ambient light irradiance and send signals to the SCN. However, melanopsin’s role in measuring light intensity is more significant and makes melanopic irradiance an effective parameter to control the impact of light on the circadian system [56].

In mouse models, ultraviolet-sensitive cones have a role in contributing to circadian entrainment and sleep–wake regulation [57]. S-cones contribute to light-evoked activity in the PON (Pretectal Olivary Nucleus), important for the light pupil reflex, and also seems to facilitate ipRGC response arrest after lights off in rodents [58]. There is conflicting evidence for the role of S-cones in humans, with one study having found no role for S-cones in NIF neuroendocrine and alerting responses [59], but a further study has found that S-cones do contribute to melatonin suppression [60]. This may indicate that the role of S-cones in melatonin suppression depends on the specific characteristics of the light exposure, such as its spectral composition or duration. S-cones may contribute to up to one-third of the response if exposure lasts ~30 min [60], while melanopsin photoreception would exclusively drive the response with ~90 min exposure [59]. Overall, there is still a debate about the relative contributions of rods, cones and melanopsin photoreception to the various NIF functions of light. However, ipRGCs are the only cells through which light affects NIF functions. In other words, if ipRGCs are blocked or removed, no NIF impact of light can be triggered [42]. 

## 5. Light: Circadian and Acute Impacts

In humans, cognitive performance remains relatively stable in well-rested individuals during the waking day. However, cognitive performance declines sharply if wakefulness is further extended into the biological night [61]. This non-linear change results from the interplay between the circadian system, temporally organising physiology and behaviour, and sleep homeostasis, keeping track of time awake and the building up of sleep need. Disturbances to the fine-tuned interplay between both systems, such as jetlag, shift work or partial sleep loss, result in cognitive impairment [61]. Light is the primary environmental cue entraining the SCN, and the circadian phase can be altered depending on its timing [62]. Light delivered in the evening and at night, up to the minimum of core body temperature (i.e., around 6 a.m., in individuals with a standard ~11 pm–7 am sleep schedule), delays the circadian phase; morning light, following the core body temperature trough, advances circadian phase. The phase-shifting impact of light has been proven with monochromatic blue (~460 nm) or polychromatic, blue-enriched light sources, but when compared with standard polychromatic bright white light sources of similar photon density, both similarly advance or delay the circadian phase [63,64,65,66]. Light can therefore have an indirect impact on alertness, sleep, and cognition through phase shifting of circadian rhythms.

Light exposure can also have acute NIF impacts on alertness, sleep, and cognition, all with a sensitivity shifted toward shorter wavelength light (~460 nm) [5,67,68,69,70,71]. Though, it should be noted that the acute NIF effects of light may not be due to a direct result of melatonin suppression through melanopsin-ipRGC suppression [72]. Light impact on alertness has been measured with subjective and objective measures with both kinds showing that light exposure increases alertness. Light exposure reduces alpha, theta, and low-frequency activity, which are correlates of sleepiness [69,73]. Furthermore, light exposure also reduces the incidence of slow eye movements, which are indicators of inattention that increase in response to sustained wakefulness, especially during the biological night [73]. Electroencephalogram (EEG) correlates of alertness are more affected by blue (460 nm) light exposure than longer-wavelength light or darkness [69,70,71]. Furthermore, a study using a custom visual display unit that could vary melanopic-irradiance found that melatonin and subjective sleepiness scores were modulated after evening exposure in healthy participants [74]. The impact of light on alertness has not been always consistently shown during the day [75,76,77,78] and may depend on the experimental context (participants laying down and/or maintained in dim light or darkness before experimental light exposure and/or sleep loss) and light parameters (duration, intensity, and spectrum). A recent meta-analysis suggests that subjective and objective measures of alertness are improved by light exposure, with subjective alertness being improved by light exposure during both the day and night. Light sources with a higher correlated colour temperature (CCT), therefore more blue-enriched light sources, appear to be more effective at modulating alertness than light sources with a lower CCT [79]. A further systematic review concludes that short wavelength light and high-intensity white light exposure influence alertness, but this depends on certain factors such as time of day [80]. 

In rodents, ipRGCs were reported to directly favour sleep during light exposure, but they also promote alertness during darkness, i.e., the absence of light is signalled by ipRGCs [81,82]. Translation of the latter finding to humans, where ipRGCs would favour sleep during darkness, is difficult to assess. However, one study in humans reported there was reduced performance in a vigilance task when participants were pre-exposed to red (635 nm) light, which could putatively be equivalent to darkness ipRGC signalling [83]. IpRGC output was also found to directly affect sleep homeostasis response to sleep loss in rodents [82]. In line with this, blue-enriched light was reported to affect sleep homeostasis in humans, most likely acting through the ipRGC pathway [70,71].

Beyond the modulation of alertness and sleepiness, light can also acutely improve cognitive performance [5] typically within 30 min (being the typical time resolution of the experiments) at night [69,73,84] and during the day [85,86]. However, as for alertness, daytime impacts are not consistently reported [75,77,78]. The performance-enhancing effects of light on cognitive functions have been shown for visual search, digit recall, serial addition–subtraction, two-column addition, logical reasoning tasks, letter cancellation tasks, and simple reaction time tasks [5,84,85,86]. Blue (470 nm) monochromatic light exposure caused a higher amplitude level on the P300, an event-related task, when compared to other monochromatic light sources [87]. There is a need for further research on how light exposure impacts cognitive functions; a systematic review reported that improvement in cognitive performance by light may depend on the spectral composition of the light, the time of day, and task complexity [80].

In rodent models, light has been reported to affect memory, and this performance impact of light on memory is mediated by ipRGCs and rod/cone photoreceptors [88,89]. Further research in rodents identified that the spatial-memory-promoting effects of light treatment are mediated by a visual circuit involving the vLGN/IGL, nucleus reuniens, and the hippocampus [90]. A resting-state fMRI study in humans during the daytime has shown that 30 min of blue (469 nm) light exposure can increase brain connectivity within networks associated with working memory and attention [91]. Longer exposure (~8 h) to blue-enriched light during the daytime also leads to improved working memory, procedural learning, and processing speed in sleep-restricted young adults [92]. Another study reported that long daytime exposure (~10 h) to high melanopic content, blue-enriched white LEDs led to an improvement in daytime cognitive function, which may not be due to changes in daytime alertness [93]. However, further research in humans is needed to understand how light can affect alertness and cognition during the day and how it impacts memory during its encoding, consolidation, and retrieval phases in humans [94]. 

## 6. NIF Brain Circuits of Light, Impact on Cognition and Inter-Individual Variations

The brain pathways of ipRGC signalling are extensively investigated in animal models [44]. Melanopsin-expressing ipRGCs (mainly M1 and M2 subtypes) project via the retinal hypothalamic tract to numerous subcortical and cortical areas of the brain, including the SCN and OPN, upstream of the Edinger–Westphal nucleus, driving pupil constriction [45]. IpRGCs innervate the ventro-lateral preoptic nucleus (VLPO), subpara-ventricular nucleus, and lateral hypothalamus, involved in sleep–wake regulation [48,95]. They also project to the amygdala and the perihabenular region [89] involved in emotional responses and mood. IpRGC efferences reach the upper brainstem superior colliculus, notably controlling eye movement, and are involved in attention [96]. IpRGCs also reach the thalamus in the intergeniculate leaflet and the pulvinar, a crossroad between cognition, attention, and alertness [97], as well as in the LGN [44]. 

The SCN has multiple direct and indirect projections to key brain regions for sleep–wake regulation such as the VLPO, paraventricular nucleus of the hypothalamus, dorsomedial nucleus of the hypothalamus, locus coeruleus, and the pineal gland, which secretes melatonin [98]. Therefore, environmental light information can be conveyed directly by the widespread projections of ipRGCs to subcortical brain regions, but also indirectly through modulating the SCN and its downstream targets. These widespread projections underlie the multiple NIF and visual functions of ipRGCs. Apart from a few studies in primates, most of these projections have been identified in laboratory mouse lines. However, these are nocturnal animals; most often they are devoid of melatonin and have their own cognitive abilities [44]. Translation to humans is therefore not straightforward.

Neuroimaging the impact of light on NIF cognitive functions in humans provides insight into the brain regions involved beyond the first retinal projections. First, a positron emission tomography (PET) study and a functional magnetic resonance imaging (fMRI) experiment investigated the impact of polychromatic white light exposure on cognitive activity during an attentional task during the day and at night. These studies demonstrated an association between light exposure and enhanced responses to the attentional tasks in the thalamus pulvinar, as well as in cortical areas [99,100].

Several fMRI studies of the NIF impacts of light followed these initial investigations. Studies using blue monochromatic light sources proved that the effect of polychromatic light modulation on brain activity, as seen in the PET and fMRI studies, was mostly dependent on blue-wavelength light, as compared to other longer-wavelength light sources [101,102,103,104,105,106]. Further light fMRI studies looked at working memory or emotional processing tasks. These studies found that brain activity increased in the thalamus, hippocampus, and amygdala regions, as well as in the prefrontal, parietal, temporal, and insular regions involved in the ongoing cognitive process in response to light [5]. In other studies, aspects of cognition such as working memory and emotional anticipation were found to be modulated after the ending of a blue-wavelength (469 nm) light exposure period (up to 40 min after 30 min of light exposure) [107,108]. This lasting effect of blue-wavelength (496nm) light was also reported to be associated with enhanced neural efficiency on the Multi-Source Interference Task, which is a complex cognitive task when compared to amber light exposure [109]. 

FMRI studies that reduced blue-wavelength (473 nm) light exposure to less than a minute indicated that subcortical areas appeared to be first affected by blue-wavelength (473 nm) light while performing an executive task with increased activity in the pulvinar, thalamus, and brainstem, as well as the amygdala, in an emotional context [101,104]. Still using short light exposure (30 s), a recent study further supported that amygdala activity was affected by light. Amygdala activity appeared, however, to be suppressed during exposure to warm long-wavelength enriched light (2800 K) [110]. This apparent discrepancy may arise from protocol and data processing differences, and in particular, the fact that participants were not engaged in any cognitive process (i.e., resting-state fMRI recordings).

Beyond its spectral quality, the impact of light on cognition appears to depend on the circadian phase and homeostatic sleep pressure. The impact of blue (473 nm) light on brain responses to a working memory task was stronger in the morning, particularly after sleep deprivation, compared to the evening a few hours before habitual sleep onset [103]. Importantly, light does have an impact on alertness, sleep, and cognition in the evening, which may be dependent on its spectral content with LED blue-enriched screens having a greater impact than non-LED screens, though the study only included male participants [111]. The modulatory effect of sleep homeostasis on the NIF impact of light on cognitive brain function is further reinforced by investigation in individuals with different variable-number (4 or 5) tandem-repeat in a portion of the PERIOD3 gene, a polymorphism associated with differences in sleep homeostasis, and vulnerability to sleep loss. Individuals homozygous for the 5-repeat genotype (PER35/5), most vulnerable to sleep loss, showed more light-induced increases in ongoing cognitive brain activity, putatively, as if the light was able to rescue part of the sleep-loss-induced changes [112].

Aside from sleep homeostasis and the circadian phase, ageing and sex may contribute to variability in the NIF impact of light. A study assessed the association between ageing and light sensitivity. Ageing was found to reduce the NIF impacts of blue-enriched light on melatonin secretion, slow-wave activity, subjective sleepiness, and sustained attention when comparing blue-enriched and non-blue-enriched polychromatic lights in young and old populations [113]. Healthy older individuals showed a reduced impact of blue (480 nm) monochromatic light on executive brain response compared to younger individuals, and this difference was not fully accounted for by the difference in age-related lens opacification [106]. 

The latter opacification can ultimately lead to the development of cataracts, which is another aspect of ageing that may affect light’s impact on the NIF system. There are contradictory findings about the benefit of implanting blue-filtering lenses for cataract surgery. Compared to older individuals with natural lenses, individuals implanted with novel lenses because of cataracts were found to show a larger impact of light on cognition and sleep [114]. In line with this, a resting-state fMRI study showed that alteration in blue light transmittance, through the implantation of blue-filtering lenses, can improve NIF responses such as alertness [115]. In contrast, the impact of light on fMRI brain responses to a working memory task was found to be similar in individuals with natural lenses or with novel lenses following cataract surgery [116]. Discrepancies between studies may arise from the delay between the experiment and the surgery, which was longer in the latter study, potentially suggesting that there was a slow adaptation of the NIF impact of light over the time period. 

Recent research has highlighted the importance of individual differences in light sensitivity, with individual traits including age, sex, chronotype, genetics, and ethnicity likely influencing individuals’ sensitivity to light. Given that individuals in industrial societies spend an increasing amount of time indoors under artificial light, it is important to understand inter-individual differences for the development of lighting recommendations and effective individually targeted integrative lighting products [117]. To close the knowledge gap of inter-individual differences, researchers have proposed key steps for the future and key research questions that need to be addressed [118].

**Figure 3:**
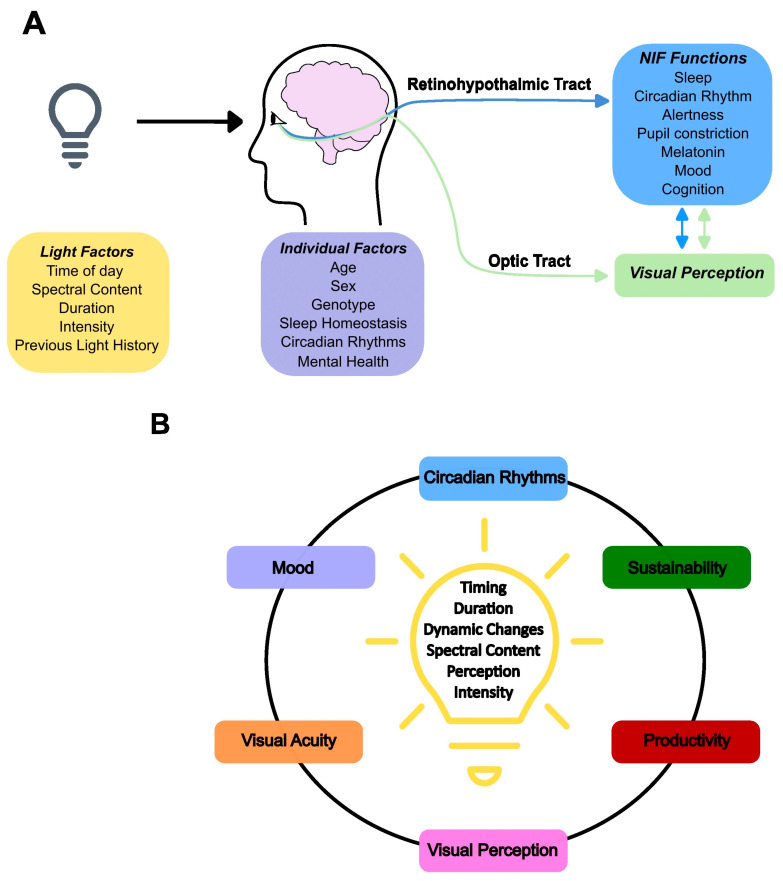
Light’s impact image forming (IF) and non-image forming (NIF) pathways. (**A**) Light signal reaches the central nervous system via the retinohypothalamic and optic tracts of the optic nerve to affect IF and NIF functions. Light impact on NIF functions depends on light factors and individual factors. (**B**) The industrial concept of integrative lighting aims to design individually tailored dynamic lighting accounting for visual perception and acuity, together with light’s impact on NIF functions, including mood, circadian rhythms, productivity (i.e., attention/alertness), and environmental sustainability. NIF’s consideration of integrative lighting largely lacks a strong scientific basis.

Collectively, the findings demonstrate that light and particularly its blue wavelength content can impact NIF brain functions (Figure 3A), and inter-individual differences play a role in light sensitivity. The mechanism of light’s impact most likely first involves the activation of subcortical brain regions that can then affect cortical activity based on the ongoing cognitive process. A detectable performance change could occur if the light’s impact is strong and/or long enough. This scenario should be verified and refined through higher-resolution neuroimaging. The recent advent of ultra-high field (UHF) MRI at 7 Tesla opens access to new spatial scales with functional studies at ~1 mm directly linked to direct structural observations at the sub-millimetre scale (0.02–1 mm) and inferences about microscopic properties (<0.02 mm; e.g., myelin content and neurite density) [119,120]. UHF-MRI will help resolve, for instance, the particular case of the impact of light on the hypothalamus in humans and especially on the SCN. An initial PET study suggested a reduction in the impact of light on the hypothalamus, over a region encompassing the SCN, after exposure to light [99]. A recent 7T fMRI study further reported reduced activity in an anterior part of the hypothalamus encompassing the SCN during exposure to different monochromatic light conditions [121]. While research in nocturnal rodents reported a decrease in SCN activity following light exposure, in line with the PET study, it shows that SCN activity is increased during light exposure, in contrast to the 7T MRI study [122]. Future research will therefore have to segregate the response of the numerous light-sensitive nuclei of the hypothalamus in humans. 

## 7. Light’s Influence on Human Cognition Is Mediated through Melanopsin Photoreception

Activation of ipRGCs using chemogenetics in mice revealed many of the direct functional targets of ipRGCs [123]. However, isolating each retinal photoreceptor’s influence on NIF functions in humans is more difficult than in animal models, as genetic and molecular techniques are not available. Therefore, the evidence for the role of melanopsin-expressing ipRGCs in NIF responses, including cognitive brain activity, has been inferred indirectly. Aside from colour-blind individuals [27], rare completely blind individuals with no functional rods and cones but who still display intact NIF responses have constituted a unique human model to isolate ipRGCs’ intrinsic photoreception [26]. Despite their complete lack of vision, these individuals have some awareness of light and can correctly guess the presence of blue (480 nm) monochromatic light exposure when presented in a two-alternative forced-choice task [124,125], potentially because of a reduction in EEG alpha power over the occipital cortex [105]. Subjective sleepiness and EEG correlates of alertness also appear to be improved with blue (480 nm) monochromatic light exposure [125]. Functional imaging of these individuals found that exposure to blue (480 nm) monochromatic light increases pulvinar and cortical activity related to ongoing executive activity [124]. More recently, an fMRI study compared healthy controls to a group of patients suffering from Leber’s Hereditary Optic Neuropathy, a disease characterized by RGC degeneration but with a relative sparing of ipRGCs. When compared to the healthy control participants, blue (480 nm) relative to red (620 nm) monochromatic light exposure increases activity over the occipital cortex in patients. Similarly, brain responses to an executive working memory task were larger in patients over the frontal cortex compared to control participants [126].

Further neuroimaging studies in healthy volunteers (i.e., no potential bias can arise from pathology) aimed at isolating melanopsin-ipRGCs’ impact on NIF brain functions. An initial study based its protocol on melanopsin bistable properties and aimed to show that prior light exposure to longer-wavelength light would increase the impact of the subsequent light exposure, as it would presumably regenerate melanopsin to its photo-transducible form. The findings were in line with this assumption as pre-exposure to orange light (~590 nm) increased the subsequent impact of a test light on prefrontal and pulvinar executive response [127]. This implied that prior light history, or photic memory, can influence the NIF impact of light on cognitive brain activity. Other fMRI studies used metameric light stimuli to isolate ipRGC-driven brain activations. Metameric light sources vary light wavelength composition to stimulate a single photoreceptor type while keeping relatively constant the stimulation of the other photoreceptors [128,129]. Melanopsin-geared metameric light stimulation led to increased cortical activity in the frontal eye field region, part of the ventral visual field during a simple dot-fixation task [129]. In addition, and still using metameric light exposure, melanopsin-geared light flickers < 0.5 Hz in four participants led to significant fMRI signal change over the occipital cortex [130], while flicker ≥ 0.5 Hz in three participants failed to do so [131]. This is presumably in line with the sluggish response time of ipRGCs. Further studies, using metameric light sources with other more cognitively demanding tasks, may elucidate wider brain activations directly dependent on melanopsin photoreception. Although many studies have reported increased brain activity in regions involved in cognitive control, whether this increase extends to the behavioural level is still under debate [132].

A few recent studies investigated the impact of continuously variating or dynamic light on NIF responses. They report that dynamic light as compared to static light may be more efficient in triggering NIF responses, as indexed through melatonin suppression and objective sleep measures [133,134]. Another study found dynamic indoor lighting at the workplace during the daytime advances melatonin onset and peripheral heat loss in the evening, which can be beneficial for people with delayed circadian rhythms [135]. Further, dynamic lighting can be also beneficial for circadian adaptation to shifted sleep–wake schedules [136]. The respective roles of spectral and illuminance changes cannot be discriminated yet, in terms of the beneficial effects of dynamic light. However, it is interesting to note that their interplay seems to be quite well-captured in the measure of melanopic equivalent daylight illuminance [137,138], further reinforcing the idea of a prominent role of ipRGCs for NIF functions and warranting further research on dynamic light’s impact on cognitive brain function, alertness, and sleep.

## 8. Emotional Processing and Mood

It is established that light can affect mood, and how our modern light environment impacts our mood needs to be carefully considered. LEDs have been beneficial in clinical settings for bright light therapy, which is used to treat seasonal and non-seasonal depressive disorders, demonstrating that light can modulate mood over long periods of time [139,140]. Seasonal associative disorder (SAD) depressive episodes are believed to be triggered by the seasonal shortening of daylight hours, as supported by its higher prevalence at higher latitudes [141]. SAD patients were also reported to show a different impact of blue and green monochromatic light in the hypothalamus in an emotional task during winter [142]. Altered light modulation of emotional processing may therefore play a role in SAD aetiology, together with retinal dysfunction and inappropriate circadian entrainment [143,144]. Healthy human beings show seasonal changes in cognitive brain responses [145], which may contribute to the cognitive impairments reported in individuals suffering from SAD [141] and to the known seasonality in the symptoms of several other psychiatric disorders [146].

Aberrant light in the evening may be particularly detrimental to mood, as shown in rodent models [9]. Light can delay the circadian timing system when administered in the evening, so evening light could contribute to suboptimal circadian entrainment, as found in SAD [143,144]. As most human beings have a circadian period slightly longer than 24 h [147], morning light is needed to advance the clock and favour earlier sleep times, and so morning light is typically considered beneficial. Whether more light in the morning can rebalance excessive evening light exposure to improve mood, sleep, and well-being is currently under investigation [148].

IpRGC photoreception is highly likely to contribute to the therapeutic effect of light exposure. Firstly, the spectral composition of light changes over the seasons, with more blue light in the summer compared to the winter [149]. Secondly, despite contradictory results about the efficacy of blue-light therapy in the treatment of seasonal and non-seasonal major depressive disorders, some studies reported that blue-light therapy, including using LEDs, is an effective treatment for SAD, but importantly requires lower irradiance and/or shorter exposure duration than standard white-light therapy [140,150,151,152], which may favour treatment compliance. Thirdly, certain individuals with genetic mutations within the melanopsin gene have an increased risk of SAD [153]. Finally, mice lacking melanopsin do not show depressive behavioural traits seen in wild-type animals exposed to aberrant light in the evening [9].

Furthermore, retina-brain pathways, which mainly involve melanopsin-ipRGCs, have been reported to be involved in light impacts on mood: the SCN-dependent pathway and the SCN-independent pathways [154]. Recently, M4-ipRGC subtypes have been implicated in a multi-synaptic pathway reaching the habenula and involved in mood regulation independently of the SCN and therefore circadian entrainment. At least in mouse models, this may be one of the pathways that are involved in the antidepressant effects of light [49]. Whether a similar functional pathway exists in humans is not known, but a neuroimaging study has found a modulation of the habenula in response to changes in luminance with a time-of-day effect [155]. The studies discussed above provide evidence for the involvement of melanopsin-ipRGCs in emotion and mood regulation. A theoretical model has been proposed for the integration of Beck’s cognitive model with light-sensitive neural circuits that are part of the emotional processing systems in the brain. Key ipRGC brain circuits include the involvement of ipRGC-hypothalamic regions and the pituitary and pineal glands, ipRGC-limbic regions, and ipRGC-thalamic regions that may underline the anti-depressant effects of light. The proposed model will help with more targeted brain research on the anti-depressive effects of light [156].

Functional imaging studies have revealed the critical roles of ventromedial and dorsolateral PFC, which have opposite activities, in depression [157]. PET studies showed that glucose metabolism in the ventromedial part of the prefrontal cortex (vmPFC), including the subgenual anterior cingulate cortex and orbitofrontal gyrus, is higher in depressed patients compared to healthy subjects [158,159]. A similar result was reported for brain activity in the vmPFC using resting state fMRI. Antidepressants can therefore help patients recover by affecting the PFC activity, and it has been shown that antidepressants are associated with decreased activity in vmPFC [160]. Recently, an fMRI study has reported reduced PFC activity (including the subgenual anterior cingulate cortex and orbitofrontal gyrus) in response to light as a function of luminance level. The suppressed brain activity is similar to the impact of chemical antidepressants, which could indicate the anti-depressive role of light in the PFC subregions [161].

Beyond the potential role of light in mood disorders, the effect of indoor lighting on emotional perception has been investigated in a healthy population. A study focused on investigating whether specific characteristics (illuminance and CCT) of a light source can influence emotional perception; there was no significant effect of light characteristics on negativity bias during an emotional oddball task. However, lower CCT (2700 K) (but not illuminance) was associated with a decrease in an individual’s negative response bias during a face-judgement task. The results suggest that the specific characteristics of a light source may be important for instant emotional perception in a healthy population, with illuminance and CCT having different roles. This light moderation of negative bias was task-dependent though [162]. While this study highlights the potential impact of indoor light on emotion, overall, the research on light’s (daylight and electrical) effect on light impressions and subjective mood states remains inconclusive [163].

## 9. Adverse Impacts on Sleep and the Particular Case of Teenagers

A quick calculation using the freely available Luox online tool [164] shows that, based on the same photopic lux, a white LED gives about 27% more melanopic irradiance than a fluorescent light source and about 40% more than an incandescent bulb. Since current research indicates that NIF responses occur over a log scale (e.g., [6,73]), this may result in a relatively limited increase in the biological impact of light. The timing of the widespread use of LED devices may therefore be more problematic than the increased blue content. Artificial lighting may be very problematic in the evening, particularly given the widespread use of screen devices that have allowed for activities that were previously difficult in darkness or under dim light.

Light exposure in the evening and at night significantly delays melatonin secretion and circadian phase, increases alertness [68], and disturbs subsequent slow-wave sleep and sleep homeostasis processes [70]. For individuals with late chronotypes, which are characterized by a longer circadian phase and/or shallower increase in sleep need [165], this is very likely to delay sleep time. Late chronotypes may also be more sensitive to light [166], further exacerbating the NIF impacts of evening light. Recent research shows that it is plausible that the advent of electric lighting contributed to the spreading of sleep timing across individuals in modern society, putatively by delaying sleep times, particularly in late chronotypes [167].

Teenagers may be at particular risk of the adverse impact of evening light. They naturally tend to be later chronotypes [168] and still need a lot of sleep. However, they are required to wake up early due to school times. They are also high consumers of evening light through electronic device screens. There is some evidence in teenagers that evening light delays melatonin secretion, circadian phase, and sleep, as in adults [169,170]. In another study, no significant changes in sleep measures were reported, however, when teenagers were exposed to a short period (1 h) of screen use before habitual bedtime [171]. Studies focusing on teenagers remain scarce, making it difficult to draw concrete conclusions about the NIF effects of light in this age group. Manipulating light exposure, particularly the timing of light exposure, is nevertheless being recommended as a potential intervention aimed at improving sleep in teenagers [170]. Importantly, it seems that imposing early restriction times on the use of screen devices in teenagers while not requesting any changes in ambient light arising from other light sources, favours earlier sleep times [172]. This finding may be associated with reduced exposure to blue-enriched LED screen light and may also have to do with the (social media) activity associated with LED screen exposure. In other words, light per se may not be the only factor curtailing sleep, but also what light allows one to do in the evening. The impact of light exposure on teenagers is a unique situation, and we have only briefly touched upon the subject here. Physiological and environmental factors most likely contribute to the sleep–wake changes seen in developing adolescents. How light environments (e.g., devices used in the evening, school and home lighting, etc.) exacerbate the changes seen in the sleep–wake cycle during adolescence is still being researched (see review [168]).

## 10. Health and Lighting

The term “blue light hazard” (BLH) is used to describe the ophthalmic phenomenon where there is potential photochemical damage caused to the retinal tissues of the eye by short wavelength light [173,174]. The potential damage from the BLH region is particularly prominent for prolonged and/or intense exposure to wavelengths < ~440 nm, especially when arising from relatively focal light sources. The BHL region is therefore distinct from the NIF impacts of diffused light, which have a peak around 460–480 nm wavelength. While there is evidence that prolonged reduction of blue wavelength content of a light source (e.g., through blue-light blocking filters) reduces photochemical damage to the retina in rodents [175,176], there is no evidence to support that exposure to blue light from LEDs increases the risk of photochemical injury for humans under normal exposure conditions. The relationship between LEDs and long-term adverse effects is still not conclusive; there is evidence of an association between age-related macular degeneration and sunlight, but whether this extends to artificial light sources is unknown [177,178]. Studies assessing LED screen devices and low-energy light bulbs have found no evidence of the blue-light hazard exposure limits [179,180].

The position of the Commission Internationale de l’Eclairage is that there is no risk of damage to the retina from the BLH hazard region from LEDs or white-light sources in general during normal use. However, there should be increased caution when exposed to optical radiation that approaches the BLH exposure limit that occurs for many days and with a continuous period of exposure [181]. A special concern may also be required with certain groups; for instance, it is recommended that blue light is not used for children’s devices, as it may be too bright [181], and there is evidence that blue light transmission through the lens changes with advanced age [106,116]. One should also avoid staring at the sun for more than 0.5 s, as this can cause solar retinitis: a type of damage that is naturally avoided by the eviction reflex of closing the eye against bright light [182].

Apart from BLH, solar retinitis, and the potential negative impact on mood reviewed in a previous section, exposure to light has been linked to other negative outcomes. IpRGC photoreception has been associated with photophobia in migraines, and therefore blue light should be used with caution in individuals suffering from migraine episodes [183]. The association between artificial light at night and cancer risk has also been studied, but the results from studies are inconclusive due to limitations with accurately assessing light exposure [184]. However, two case-control studies assessed exposure to light using satellite images and were able to differentiate light wavelengths. The studies found outdoor light in the blue spectrum was positively associated with an increased risk of breast, prostate, and colorectal cancers [185,186]. Artificial light is a modifiable cancer risk factor and therefore a better understanding of the association between artificial light at night and cancer is needed, and it is important for developing recommendations for the use of artificial light at night [184].

Furthermore, digital eye strain refers to eye problems caused by the prolonged use of digital devices, including eye strain, dry eyes, blurred vision, headaches, and neck pain. Currently, the evidence to support the use of blue-blocking lenses and filters for digital eye strain is inconclusive and more randomized controlled trials are needed [187,188,189]. Finally, visual acuity also appears to be affected by focusing on screen devices at a close distance and for a prolonged time, raising concerns about a predicted increase in myopia, though there are many other risk facts involved in myopia development [190]. While time spent outdoors has been seen to have a protective effect on myopia onset [191]. There is evidence from mice studies that ipRGCs have a role in myopia progression and ocular growth [192]. However, the exact impact of screen-emitted light on visual acuity still needs to be thoroughly assessed [190]. Furthermore, it has been proposed that prolonged exposure to LEDs may prompt myopia development through disruption of retinal circadian rhythms. Research in animal models supports a negative link between LEDs and the disruption of retinal circadian rhythms and mammalian refraction development. More research is needed in humans, as currently there is only circumstantial evidence of this link [12].

Overall, there is concern about the potentially harmful effects of blue light that is increasingly available in white LEDs, e.g., through LED screen devices, but also for medical purposes [174,193]. However, the increase in blue light in LEDs is unlikely to be the main driver of health issues; other key factors need to be taken into account when discussing health issues surrounding lighting, including sleep–wake schedules, circadian rhythms, duration of screen use, evening and late-night use of light sources and screen devices, and repeated long-term exposure. Here, we have briefly highlighted some of the impacts of light on human health, but light may potentially have a much broader influence on human health (see review [10]). Understanding the role of light in health and well-being needs to be placed in context, as many other factors need to be considered when discussing light’s influence on human health [10]. Whilst there is clear evidence that light does impact health and well-being, research still needs to establish how to optimize the prevention of the negative impacts of inappropriate light while maintaining visual functions and favouring positive NIF effects.

## 11. Light Environments

Studies are increasingly looking at how altering light environments in the “real world” may improve health and well-being. Optimizing lighting with blue-enriched light sources in offices had a beneficial impact on subjective alertness, mood, performance, and sleep in comparison to standard lighting [194]. Classrooms with blue-enriched light sources were associated with a beneficial impact on cognitive performance in students [195]. Likewise, blue-enriched light treatment can improve sleep quality and cognitive function in Alzheimer’s patients [196,197]. In patients with disorders of consciousness (DOC) that still show detectable signs of a sleep–wake cycle (this is not the case in many DOC patients), blue-light treatment in the morning in combination with caffeine and melatonin treatment caused an improvement in sleep and circadian rhythms [198]. A further study looked at long-term (3.5 years) exposure to daily polychromatic light (~1000 lux) in combination with or without melatonin in multiple care facilities. In the bright light condition without melatonin, there were reduced cognitive deficits, improvements in depressive symptoms, reductions in increasing functional limitations, and improvements in sleep duration over time in the elderly. Furthermore, in combination with melatonin, bright light exposure improved aspects of sleep that improve over time with the treatment. Further long-term studies on light and/or melatonin will help to determine effects that develop slowly and have previously been missed in short-term studies [199].

Given that LEDs can be tuned almost infinitely, LED lighting has the potential to play a major role in promoting health and cognition. The concept of integrative lighting (traditionally referred to as “human-centric lighting”) developed out of these new possibilities. Integrative lighting aims to take into account all the visual and NIF impacts of light to dynamically change light spectral content and intensity over the day, with a potential benefit for cognitive performance, sleep regulation, emotion, mood, and well-being (Figure 3B) [13,16]. Considering the NIF effects, recommendations were recently proposed for indoor lighting during the daytime, evening, and nighttime [200].

Research on dynamic lighting is becoming more common; however, the number of studies is still relatively low. Currently, studies have produced mixed results with the main reported benefit of dynamic lighting being sleep-related effects due to increased light levels during the day. This may in part be due to different theoretical aims of studies, protocol differences, and different lighting scenarios [201]. Certain studies have also highlighted the sleep-related benefits of dynamically changing light spectra for hospitalized patients [134,202]. A recent study looked at the impact of dynamic lighting over a longer time scale (48 h) on subjective wellness measures, cognitive performance, and sleep measures. Dynamic lighting compared to static lighting was found to be beneficial for sleep-related effects and there was also a beneficial impact on the other metrics, but this was dependent on a time of day and experimental day effect. The study provides evidence that dynamic lighting is beneficial to a “stimulated” office environment; however, no conclusive pattern emerged from the study. These considerations highlight the need for more research on dynamic lighting in larger data sets and the need to investigate how inter-individual differences impact responses to dynamic lighting [203]. The optimization of dynamic light is challenging because the design of dynamic lighting scenarios may be different depending on the aims (e.g., which NIF functions are being targeted) and the real-life environmental context. Depending on these factors, different dynamic lighting scenarios could be developed, but further research on dynamic lighting with larger datasets on longer time scales and outside of laboratory studies is still needed before successful implementation [201].

A study in a small number of healthy male volunteers showed that NIF responses to light, including melatonin suppression, sleep measures, and modulation of alertness and cognitive performance, can be caused by using white LED backlight screen devices in the evening, most likely due to the high short-wavelength content of white LEDs [111], as expected based on previous research using other light sources. However, the impact of year-long exposure to light in the evening and at night, including blue-enriched light is not known in humans. The knowledge gap is not new but may be even more evident now that LEDs allow for “any light, anywhere and anytime”. The success of individually targeted lighting devices will depend in part on a better understanding of the complex light-sensitive pathways of the brain and the bases of inter-individual differences in light influence on NIF physiology, including age, sex, mental health, and genotype [117]. Although field interventional studies are increasingly carried out, the translation of in-lab findings to help design field studies and interventions also remains insufficient [8,204].

Finally, as we continue to develop lighting environments that ”mimic” natural daylight, more evidence is required to understand the assumed benefits of natural daylight over electrical lighting [8]. A full discussion on natural daylight is beyond the scope of this review; however, it is important to recognize the importance of natural daylight for human health and well-being. Researchers have already established key knowledge gaps within the natural daylight field and have proposed research aims for the future [204,205]. How we continue to develop our electrical light environments in combination with our natural daylight environments is a complicated research question, where interdisciplinary research is no doubt needed to ensure the development of light environments that benefit human health and well-being.

## 12. Conclusions

We have moved away from traditional indoor lighting, which used to be of lower intensity and blue-depleted compared to natural light. White LED lighting has led to more blue-wavelength light exposure potentially closer to natural light. As individuals in industrial societies spend a large part of each day indoors under electric lighting, it is an important research question to address to better understand the NIF impacts of light.

We suggest that future research on the NIF impacts of light should focus on the following research aspects. Firstly, the exact dose of light required to impact NIF physiology is not known and how the characteristics of the light source (intensity, wavelength, duration, timing, and dynamic changes) and inter-individual differences (age, sex, and genotype) will impact the NIF functions remains to be fully elucidated. Secondly, the use of high-resolution neuroimaging in humans should refine the in vivo brain wiring of the NIF impacts of light under different cognitive tasks. Thirdly, the impact of repeated and/or long-term light exposure remains to be fully characterised. Fourthly, separating the different negative impacts of light exposure, the detrimental NIF effects on mood and sleep, and the potential reduction of visual acuity is required to optimize lighting recommendations. Finally, lab findings should be more thoroughly translated to field studies, including assessing inter-individual differences, e.g., between age groups (infants, young children, teenagers, and the elderly), and the visual roles of light, to make integrative lighting a concept truly based on scientific findings.

## Data Availability

Not applicable.

## Abbreviations

BLH	blue light hazard
CCT	correlated colour temperature
DOC	disorders of consciousness
EEG	electroencephalogram
fMRI	functional Magnetic Resonance Imaging
ipRGCs	intrinsically photosensitive retinal ganglion cells
LED	Light-emitting diode
LGN	lateral geniculate nucleus
NIF	non-image forming
OPN	olivary pretectal nuclei
PET	Positron Emission Tomography
PFC	prefrontal cortex
PLR	pupil light reflex
RGC	retinal ganglion cells
S-cones	short wavelength-cones
SAD	seasonal associative disorder
SCN	suprachiasmatic nuclei
VLPO	ventro-lateral preoptic nucleus
UHF	ultra-high field
